# miR-145 sensitizes esophageal squamous cell carcinoma to cisplatin through directly inhibiting PI3K/AKT signaling pathway

**DOI:** 10.1186/s12935-019-0943-6

**Published:** 2019-09-30

**Authors:** Tian-Liang Zheng, De-Ping Li, Zhan-Feng He, Song Zhao

**Affiliations:** 1 Department of Thoracic Surgery, The First Affiliated Hospital of Zhengzhou University, No. 1, East Jianshe Road, Erqi District, Zhengzhou, 450000 Henan Province People’s Republic of China; 2 Department of Acupuncture and Moxibustion, Zhengzhou Hospital of Traditional Chinese Medicine, Zhengzhou, 450000 People’s Republic of China

**Keywords:** Esophageal squamous cell carcinoma, Cisplatin, miR-145, Chemosensitivity, PI3K/AKT signaling pathway

## Abstract

**Background:**

Esophageal squamous cell carcinoma (ESCC) is the eighth most common cancer worldwide and is one of the most lethal malignancies. Cisplatin (DDP) is a key drug for ESCC treatment, but the presence of chemotherapy resistance limits the use of DDP. To enhance chemosensitivity to DDP is important for ESCC treatment.

**Methods:**

qRT-PCR and Western blotting detected mRNA and protein expression in ESCC tissues and cells. Luciferase reporter assay assessed the interaction between miR-145 and AKT3. Cell cycle, apoptosis and proliferation were investigated with flow cytometry and MTT assay, respectively. Nude mice xenograft model was established, and immunohistochemistry (IHC) and TUNEL assay were conducted to detect Ki-67 level and apoptosis in xenograft tumor.

**Results:**

Down-regulated miR-145 and up-regulated AKT3 were observed in ESCC tissues and cells. Luciferase reporter assay revealed that miR-145 negatively regulated AKT3 through binding to its 3′-UTR. Overexpression of miR-145 or knockdown of AKT3 promoted DDP-induced cell cycle arrest and apoptosis, as well as reduced IC50 of DDP treatment, which was reversed by AKT3 overexpression. The expression level of MRP1, P-gp, CyclinD1, c-Myc and anti-apoptotic protein Bcl-2 were down-regulated, while pro-apoptotic protein Bax was up-regulated by miR-145. Furthermore, overexpression of miR-145 enhanced the DDP-induced tumor growth suppression in vivo.

**Conclusion:**

miR-145 increased the sensitivity of ESCC to DDP, and facilitated DDP-induced apoptosis, cycle arrest by directly inhibiting PI3K/AKT signaling pathway to decrease multidrug resistance-associated proteins MRP1 and P-gp expression. Improving the efficacy of DDP by boosting the miR-145 level provides a new strategy for treatment of ESCC.

## Background

Esophageal squamous cell carcinoma (ESCC) is the eighth most common cancer worldwide, especially in Asia, and its mortality ranks the second in cancer-related deaths [1]. Smoking, alcohol, and environmental factors are major contributors to ESCC [2]. Although the treatment of ESCC is mainly based on surgical resection, therapies that combines surgery with radiotherapy, chemotherapy, and chemoradiotherapy are also utilized [3]. Nowadays, cisplatin (DDP) and 5-Fluorouracil (5-FU) have been accepted as the first-line drug for ESCC treatment in many countries. However, due to the chemotherapy resistance, current chemotherapies in ESCC are of limited efficacy [4, 5]. Therefore, it is important to explore the molecular mechanisms of chemotherapy resistance and find the key molecular targets for increasing the efficacy of chemotherapy drugs.

Drug resistance is a big issue in chemotherapies, where various proteins have been reported to play a role. Amongst them, proteins of ATP-binding cassette (ABC) transporter family are the most widely recognized ones, since they can extrude anticancer drugs out of cells to reduce the effects of chemotherapeutics efficacy. As reported, multidrug resistance-associated protein 1 (MRP1, also known as ABCC1) expression level was closely correlated with adverse chemotherapy outcomes in various cancers, including breast cancer, acute myeloid leukemia and non-small cell lung cancer [6, 7]. P-glycoprotein (P-gp, also known as ABCB1) is another protein of the ABC transporter family, with 19% similarity to the MRP1 sequence. Similarly, P-gp expression has been reported to negatively correlate with chemotherapy response in small cell lung cancer and compromised outcomes [8]. Additionally, P-gp and MRP1 are reported to highly express in ESCC tissues compared to distal non-cancerous tissues [9]. These studies indicated that MRP1 might mediate chemosensitivity in ESCC as well.

MicroRNAs (miRNAs) are a group of small non-coding RNAs with a length of ~ 22 nucleotides [10, 11]. Emerging evidences demonstrate that miRNAs play important roles in a variety of biological processes by predominantly binding to 3′-UTR regions of targeted sequences, resulting in the suppression or degradation of mRNA. In the past decades, miRNAs were clearly linked to cancer development and chemotherapy sensitivity [12]. Hummel et al. [13] summarized several miRNA signatures in ESCC cell lines demonstrating chemotherapy resistance in a recent review. miR-145 has been reported as a tumor suppressor in a variety of tumors. Previous study suggested that miR-145 suppressed cell proliferation and metastasis by inhibiting PI3K/AKT3 signaling pathway in thyroid cancer [14, 15]. Additionally, miR-145 was validated to play a vital role in modulating drug resistance in different cancer cells [15, 16]. Gao et al. [15] demonstrated that miR-145 sensitized breast cancer to doxorubicin by regulating MRP-1. Additionally, it was reported that miR-145 was significantly decreased in ESCC tissues by microRNA microarray, and miR-145 could be regarded as a potential tumor marker for diagnosis of ESCC [17, 18]. Exogenous addition of miR-145 could suppress the invasion and proliferation of ESCC cells [17]. However, the relationship between miR-145 and chemosensitivity of ESCC and its underlying molecular mechanism are still unclear.

In the present study, we found that miR-145 could increase the sensitivity of ESCC to DDP and enhance the suppressive efficacy of DDP on ESCC growth. Furthermore, miR-145 could promote DDP-induced apoptosis, cycle arrest by directly inhibiting PI3K/AKT3 signaling pathway to decrease the expression level of MRP1, P-gp and proliferation-related proteins. Exogenous increase of miR-145 level could effectively suppress the tumorigenesis and tumor growth in xenografts mice model, proving a potential strategy for ESCC treatment. miR-145 might be a promising therapeutic target for chemosensitization of ESCC cells to DDP.

## Materials and methods

### Clinical tissue specimens

30 pairs of ESCC and normal adjacent esophageal epithelial tissues were obtained from the First Affiliated Hospital of Zhengzhou University. Tissues were frozen immediately in liquid nitrogen and then stored in − 80 °C for mRNA extraction and analysis. The study protocol was designed and approved by the ethical committee of the First Affiliated Hospital of Zhengzhou University, and informed consent was obtained from all patients.

### Cell culture

Normal esophageal epithelial cell line Het-1A was purchased from Jenniobio Biotechnology (Guangzhou, China) and five esophageal squamous carcinoma cell line (EC109, EC9706, KYSE-150, KYSE-30 and TE-1) were purchased from Chinese Academy of Medical Sciences (Beijing, China). Except for KYSE-150, other cells were cultured with RPMI-1640 medium (Invitrogen, CA, USA) supplemented with 10% fetal bovine serum (Gibco, USA), 100 U/mL penicillin (Invitrogen, USA) and 100 μg/mL streptomycin (Invitrogen, CA, USA). KYSE-150 was cultured with Dulbecco’s Modified Eagle’s medium (DMEM) supplemented with 10% FBS, 100 U/mL penicillin and 100 μg/mL streptomycin. And cells were maintained in humidified incubator with 5% CO_2_ at 37 °C.

### RNA extraction and qRT-PCR

Total RNA was extracted from tissues and cells with Trizol reagent (Invitrogen, CA, USA) according to the manufacturer’s instructions. The cDNA was synthesized using the PrimeScript^®^ RT reagent Kit with gDNA Eraser (Takara, China). The relative transcription level of target genes was determined with quantitative PCR using the SYBR^®^ Premix Ex TaqII kit (Takara, China). GAPDH and U6 were used as the internal control for AKT3 and miR-145, respectively. Each reaction was performed in triplicate using Applied Biosystems Step One Plus Real-Time PCR System (Applied Biosystems, CA, USA). The relative expressions of genes were calculated by 2^−ΔΔct^. All gene-specific primers are listed as following: miR-145 forward primer: 5′-CGGTCCAGTTTTCCCAGGA-3′, miR-145 reverse primer: 5′-GTCGTATCCA GTGCAGGGTCCGAGGTATTCGCACTGGATACGACAGGGAT-3′. U6 forward primer: 5′-GCTTCGGCAGCACATATACTAAAAT-3′, U6 reverse primer: 5′-CGCT TCACGAATTTGCGTGTCAT-3′. AKT3 forward primer: 5′-AATGACTATGGCCGAGCAGT-3′, AKT3 reverse primers: 5′-ATCAAGAGCCCTGAAAGCAA-3′; GAPDH forward primer: 5′-CAAATTCCATGGCACCGTCA-3′, GAPDH reverse primers: 5′-GGAGTGGGTGTCGCTGTTG-3′.

### Western blot analysis

Cells were harvested and lysed in the RIPA buffer (Sigma-Aldrich, USA). Protein concentrations were determined using the BCA protein assay kit (Thermo Fisher Scientific, USA). Then equal aliquots of proteins were separated on 10% SDS-PAGE. Following electrophoresis, the separated proteins were transferred onto PVDF membranes (EMD Millipore, Billerica, MA, USA). Membranes were blocked with 5% bovine serum albumin in TBST and then incubated in primary antibodies overnight at 4 °C followed by secondary antibodies for 1 h at 37 °C. Primary antibodies AKT, p-AKT, Bcl-2, Bax, c-Myc, Cyclin D1 were purchased from Cell Signaling Technology (USA). MRP1 and P-gp were purchased from Abcam (Cambridge, UK). GAPDH was purchased from Sigma Aldrich (MO, USA) and used as the internal control. Finally, the signal was visualized through a chemiluminescent detection system (Pierce ECL Substrate Western blot detection system, Thermo, Rockford, IL, USA) and bands were analyzed with Image J (NIH, USA).

### MTT assay

ESCC cells were treated with 5 μM, 10 μM, 20 μM, 40 μM or 80 μM DDP for 24 h. At the end of treatment, the cell proliferation was determined with MTT assay. Cells were seeded on 96-well microplates at 1 × 10^4^ cells/well for 48 h. Then medium 20 μL of MTT assay solution was added and incubated with cells for 4 h. At the end of the incubation, plates were measured at 570 nm using a microplate reader (BioTek, Winooski, VT, USA). Each experiment has at least 3 independent tests.

### Plasmids construction and transfection

The full-length AKT3 sequence was constructed into pcDNA3.1 and the reconstructed plasmids were named pcDNA3.1-AKT3. The empty plasmid was employed as negative control. AKT3 specific short hairpin RNAs (shRNA) were cloned into pSicoR vector (sh-AKT3). The miR-145 mimics and inhibitor were synthesized by GenePharma Company (Shanghai, China). Transfection was conducted with Lipofectamine 2000 reagent (Invitrogen, CA, USA) according to the manufacturer’s protocols. After transfection for 48 h, cells were collected and used for further experiments.

### Dual-luciferase reporting assay

The 3′-UTR of the wildtype AKT3 and a variant containing mutations in the putative binding site were inserted downstream of the firefly luciferase reporter into the psiCHECK-2 vector (Promega, Madison, WI, USA). Constructed luciferase reporter plasmids (wildtype or mutant AKT3 plasmids) were co-transfected with miR-145 mimics or miR-NC into cells using Lipofectamine 2000. After 48 h, the luciferase activity was determined using Dual-Luciferase Reporter Assay System according to the manufacturer’s protocol (Promega, Madisoon, WI, USA).

### Flow cytometry for cell cycle and apoptosis analysis

Flow cytometry was utilized to detect the cell cycle and apoptosis. Cells transiently transfected with miR-145 mimics, miR-145 inhibitor or various plasmids (sh-AKT3 and pcDNA3.1-AKT3) were seeded in 6-well plates and cultured for 48 h. Then cells were collected with trypsin digestion solution, washed twice with phosphate-buffered saline (PBS). For cell cycle analysis, cells were resuspended in 200 μL PBS with 10 μL propidium iodide (PI) and incubated at room temperature in the dark for 15 min. For apoptosis analysis, Annexin V/Dead Cell Apoptosis Kit (Invitrogen, CA, USA) was utilized according to the manufacturer’s instructions. Briefly, 5 µL Annexin V-FITC and 5 µL PI was added to each well and cells were incubated in the dark for 15 min. The cell cycle and cell apoptosis were measured using the FACScan flow cytometry (Becton, CA) equipped with CellQuest Software (Becton–Dickinson).

### Nude mice xenograft experiments

The animal experiments were approved by the Ethics Committees of the First Affiliated Hospital of Zhengzhou University. Six-week-old male BALB/c nude (nu/nu) mice were purchased from SJA Laboratory Animal Co., Ltd (Hunan, China; n = 28). The tumor xenograft mice model was established as previously described with minor modifications [19]. Briefly, EC109 cells were suspended in PBS at a final concentration of 1 × 10^8^ cells/mL. Mice were divided randomly into four groups (i.e. miR-NC group, miR-145 mimics group, miR-NC + DDP group, miR-145 mimics + DDP group, seven mice per group). The mice received an intraperitoneal injection of DDP (1 mg/kg) with or without miR-145 mimics starting from the third week every 4 days. At 30 days post injection, the mice were sacrificed by cervical dislocation in diethyl ether anesthesia, and the tumors were dissected and weighed. Tumor volume (V) was calculated by the formula: V = 0.5 × length × width^2^.

### Immunohistochemistry (IHC)

Tumor tissues were fixed in 4% paraformaldehyde and washed with PBS, then transferred to 70% ethanol and embedded in paraffin. Sections were cut from paraffin blocks into 4 μm thick. The sections were dewaxed in xylene and dehydrated with gradient ethanol. After antigen retrieval in sodium citrate buffer, the sections were stained with Ki-67 antibody (cell signaling technology, USA) overnight at 4 °C. The complex was visualized with DAB complex, and the nuclei were counterstained with haematoxylin. The slices with brown or yellow cytoplasm were considered to be positive.

### TUNEL assay

Tumor tissues were excised, and the apoptosis was detected with TUNEL assay kit (Fluorescein In Situ Cell Death detection kit, Roche) according to manufacturer’s instruction. Briefly, 4 μm-tissues slides were prepared and fixed in 4% paraformaldehyde. Then slides were permeabilized in 0.1% Triton x-100 and incubated in TUNEL reaction mix for 1 h at 37 °C. The nuclei were counterstained with hematoxylin and observed under a microscope.

### Statistical analysis

Data were presented as mean ± standard deviation (SD). All statistical analyses were performed by Graphpad Prim 5 (GraphPad Software, La Jolla, CA, USA). The correlation between miR-145 expression and clinicopathological characteristics of ESCC patients was assessed by the Chi squared test. Spearman correlation analysis was performed to analyze the correlation between miR-145 and AKT3 in ESCC tissues. Student’s *t* test was employed to compare the difference between two groups. The statistical analysis between multi-groups was carried out using one-way analysis of variance (ANOVA) by Tukey post hoc test. A two-side value of p < 0.05 was considered statistically significant.

## Results

### Low expression of miR-145 and high expression of AKT3 are observed in ESCC tissues and cells

**Fig. 1:**
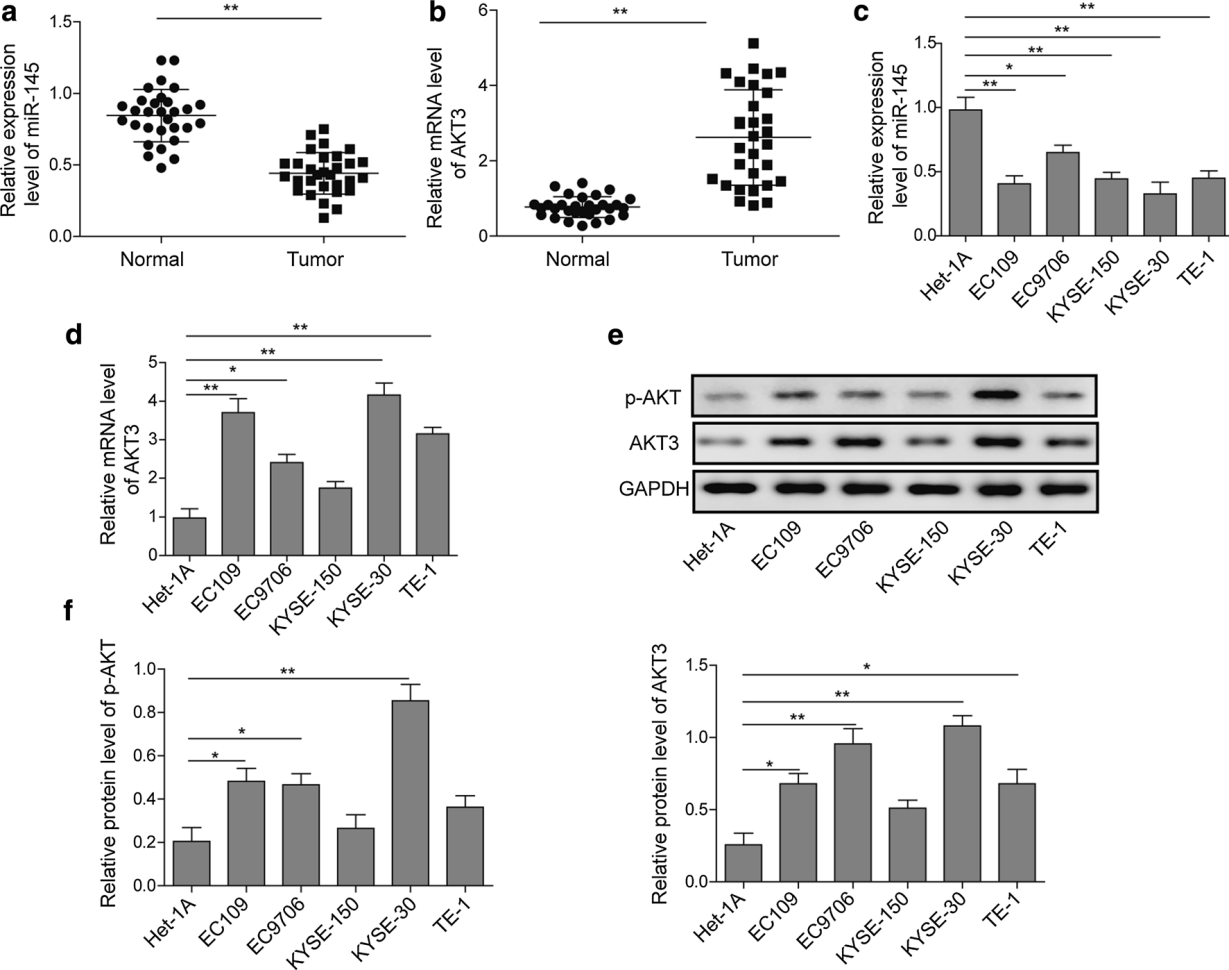
The expression level of miR-145 and AKT3 in ESCC tissues and cells. The level of miR-145 (**a**) and AKT3 (**b**) in ESCC tissue (n = 30) compared with adjacent normal tissues was detected by qRT-PCR. The expression level of miR-145 (**c**) and AKT3 (**d**) was detected by qRT-PCR in normal esophageal squamous cells line and ESCC cell lines. **e** Western blot analysis of AKT3 and p-AKT in normal esophageal squamous cells line and ESCC cell lines. **f** Quantification of relative protein level for Western blotting. Total 30 subjects were analyzed. All the results were shown as mean ± SD (n = 3), which were three separate experiments performed in triplicate. *p < 0.05 and **p < 0.01

Total RNA was extracted from ESCC and normal adjacent esophageal epithelial tissues, and subjected to the qRT-PCR to determine the expression level of miR-145 and AKT3. In tumor tissue, miR-145 was significantly down-regulated compared to the normal adjacent esophageal epithelial tissues (n = 30) (Fig. 1a). Meanwhile, the mRNA level of AKT3 was dramatically elevated in tumor tissue (n = 30) (Fig. 1b). Furthermore, clinicopathological characteristics of ESCC patients showed that there was a significantly co-relation between low miR-145 level and advanced TNM stage (Table 1). To verify the hypothesis that there is an inverse correlation between miR-145 and AKT3 expression level in ESCC, we tested the AKT3 and miR-145 expression level in normal esophageal squamous cells line (Het-1A) and five ESCC cell lines (EC9706, EC109, KYSE-150, KYSE-30 and TE-1). The data revealed that compared with normal esophageal squamous cells, the miR-145 level was down-regulated in five ESCC cells, whereas AKT3 mRNA level was significantly up-regulated (Fig. 1c, d). Then total protein was extracted and subjected to Western blot analysis and the results were consistent with qRT-PCR (Fig. 1e, f). To conclude, these data above demonstrated that AKT3 is up-regulated in ESCC tissues and cells. Because the expression of miR-145 was the lowest in EC109 and KYSE-30 cells, they were employed in the following studies.

**Table 1:** Correlation between the expression levels of miR-145 and the clinicopathological characteristics of ESCC patients

Clinical parameters	Cases (n)	miR-145 expression		P-value (*P < 0.05)
		High (n)	Low (n)	
Gender				
Male	21	9	12	0.232
Female	9	6	3	
Age				
< 60	17	7	10	0.269
≥ 60	13	8	5	
Tumor location				
Upper	7	3	4	0.753
Middle	12	7	5	
Lower	11	5	6	
Tumor size (cm)				
< 3	16	10	6	0.143
≥ 3	14	5	9	
Differentiation grade				
Well-moderate	23	12	11	0.666
Poor-undifferentiation	7	3	4	
Lymph node metastasis				
Negative	15	10	5	0.068
Positive	15	5	10	
TNM stage				
I–II	14	10	4	0.028*
III–IV	16	5	11	

### miR-145 inhibits AKT3 expression through the direct interaction with the 3′-UTR

**Fig. 2:**
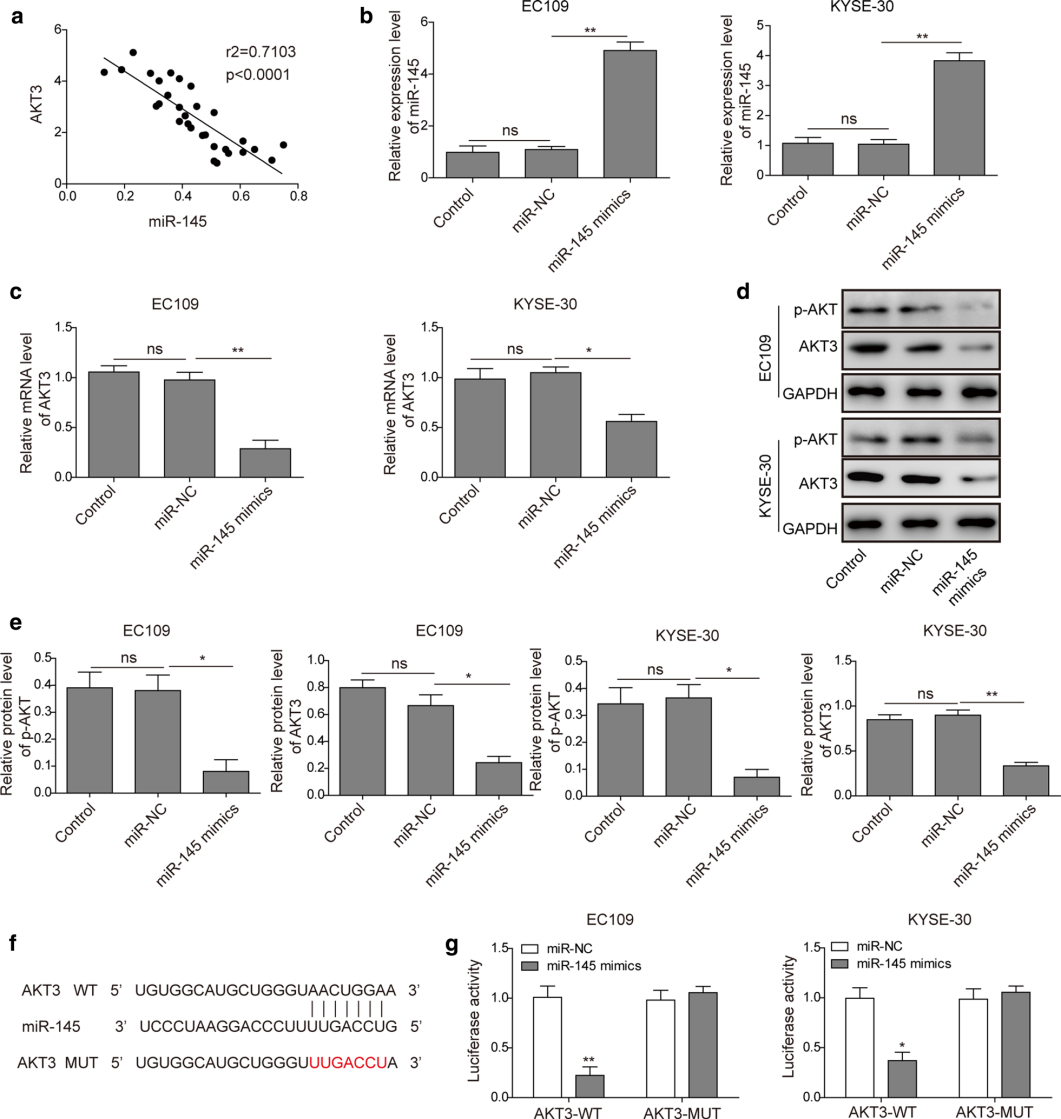
miR-145 negatively regulated AKT3 expression by binding to the 3′-UTR of AKT3. **a** Spearman’s correlation analysis of the correlation between the miR-145 and AKT3 mRNA expression level in ESCC. The expression level of miR-145 (**b**) and AKT3 (**c**) were detected with qRT-PCR in EC109 and KYSE-30 cells transfection with miR-145 mimics or miR-NC. **d** Western blot analysis of AKT3 and p-AKT in EC109 and KYSE-30 cells transfection with miR-145 mimics or miR-NC. **e** Quantification of relative protein level for Western blotting. **f** miR-145 and its putative binding sequence in the 3′-UTR of AKT3. **g** Luciferase reporter assay showed the luciferase activity in miR-145 mimics and AKT3 wild type/mutant co-transfected EC109 and KYSE-30 cells. All the results were shown as mean ± SD (n = 3), which were three separate experiments performed in triplicate. *p < 0.05 and **p < 0.01

The data of miR-145 and AKT3 were subjected to the Spearman’s correlation analysis and an inverse correlation was revealed between them (Fig. 2a). The expression level of miR-145 was significantly increased in EC109 and KYSE-30 cells transfected with miR-145 mimics (Fig. 2b). qRT-PCR results demonstrated that increasing miR-145 significantly inhibited the mRNA level of AKT3 in EC109 and KYSE-30 cells (Fig. 2c). Furthermore, Western blotting revealed that the protein level of AKT3 was significantly decreased in miR-145 overexpressed EC109 and KYSE-30 cells (Fig. 2d, e). These results suggested that AKT3 are negatively regulated by miR-145. To understand the potential target of miR-145 in ESCC, the predicted targeting 3′-UTR region of human AKT3 was mutated and cloned into the psiCHECK-2 vector (Fig. 2f, labeled as AKT3 3′-UTR mutation). In parallel, the corresponding wildtype 3′-UTR region of AKT3 was constructed as well. Co-transfecting the wildtype AKT3 3′-UTR plasmid and miR-145 mimics into ESCC cells significantly reduced the luciferase activity, which was not affected by AKT3 mutation (Fig. 2g). These results demonstrated that AKT3 is one direct target of miR-145 in ESCC, and miR-145 can inhibit PI3K/AKT signaling pathway by directly down-regulating AKT3.

### miR-145 inhibits multidrug resistance and proliferation-related signaling by directly down-regulating AKT3

**Fig. 3:**
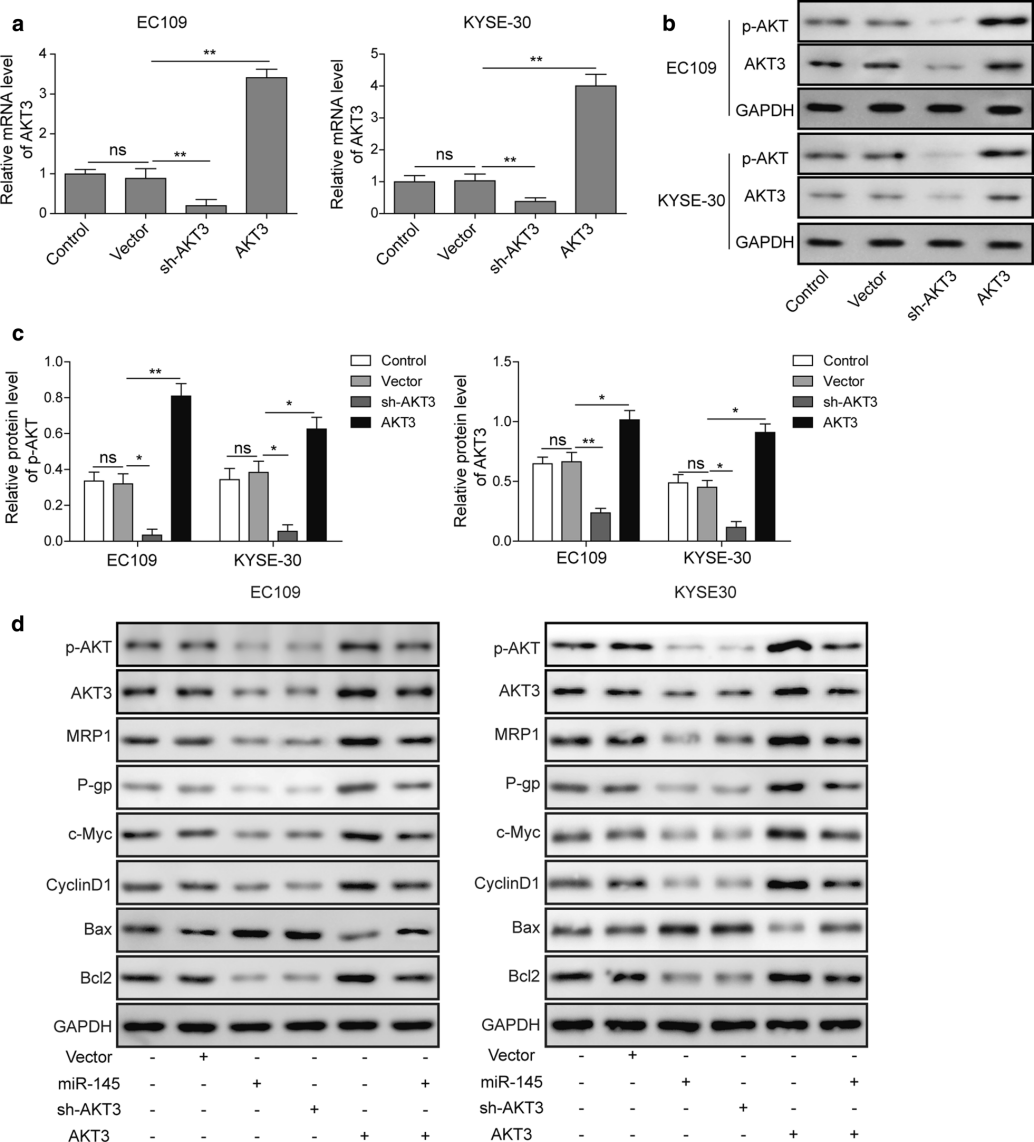
Overexpression of miR-145 inhibited multidrug resistance and cell proliferation-related signaling by down-regulating AKT3. **a** The mRNA level of AKT3 was detected by qRT-PCR in EC109 and KYSE-30 cells transfected with sh-AKT3 and pcDNA3.1-AKT3. **b** The protein level of AKT3 and p-AKT was assessed by Western blotting in EC109 and KYSE-30 cells transfection with sh-AKT3 and pcDNA3.1-AKT3. **c** Quantification of relative protein level for Western blotting. **d** The multidrug resistance and cell proliferation-related protein level was detected by Western blotting in EC109 and KYSE-30 cells transfected with miR-145 mimics, sh-AKT3 or pcDNA3.1-AKT3. All the results were shown as mean ± SD (n = 3), which were three separate experiments performed in triplicate. *p < 0.05

To investigate the effect of miR-145 and downstream signaling of AKT3 in ESCC, AKT3 overexpression and knockdown plasmids were constructed and were co-transfected with/without miR-145 mimics into ESCC cells. qRT-PCR and Western blotting demonstrated that sh-AKT3 markedly reduced the AKT3 expression, while AKT3 overexpression plasmids dramatically increased AKT3 expression compared to the vector control (Fig. 3a–c). Additionally, we measured the expression of multidrug resistance and survival-related AKT3 targets, namely MRP1, P-gp, c-Myc, cyclin D1, Bax, and Bcl-2. As shown in Fig. 3d, overexpression of miR-145 and knockdown of AKT3 could significantly reduce multidrug resistance-associated protein MRP1 and P-gp expression, while overexpression of AKT3 could reverse the above effects in miR-145-overexpressing cells. Corresponding to these changes, the pro-survival c-Myc, cyclinD1, and Bcl-2 levels were markedly reduced, while that of pro-apoptotic Bax was increased in miR-145 mimics or sh-AKT3-treated ESCC cells. The alterations on these targets were abolished by up-regulation of AKT3 in miR-145-overexpressing cells (Fig. 3d and Additional file 1: Fig. S1). These data suggested that miR-145 may regulate cell proliferation and chemosensitivity by inhibiting PI3K/AKT signaling pathway-mediated downstream target genes in ESCC.

### miR-145 sensitizes ESCC cells to DDP through inhibiting AKT3

**Fig. 4:**
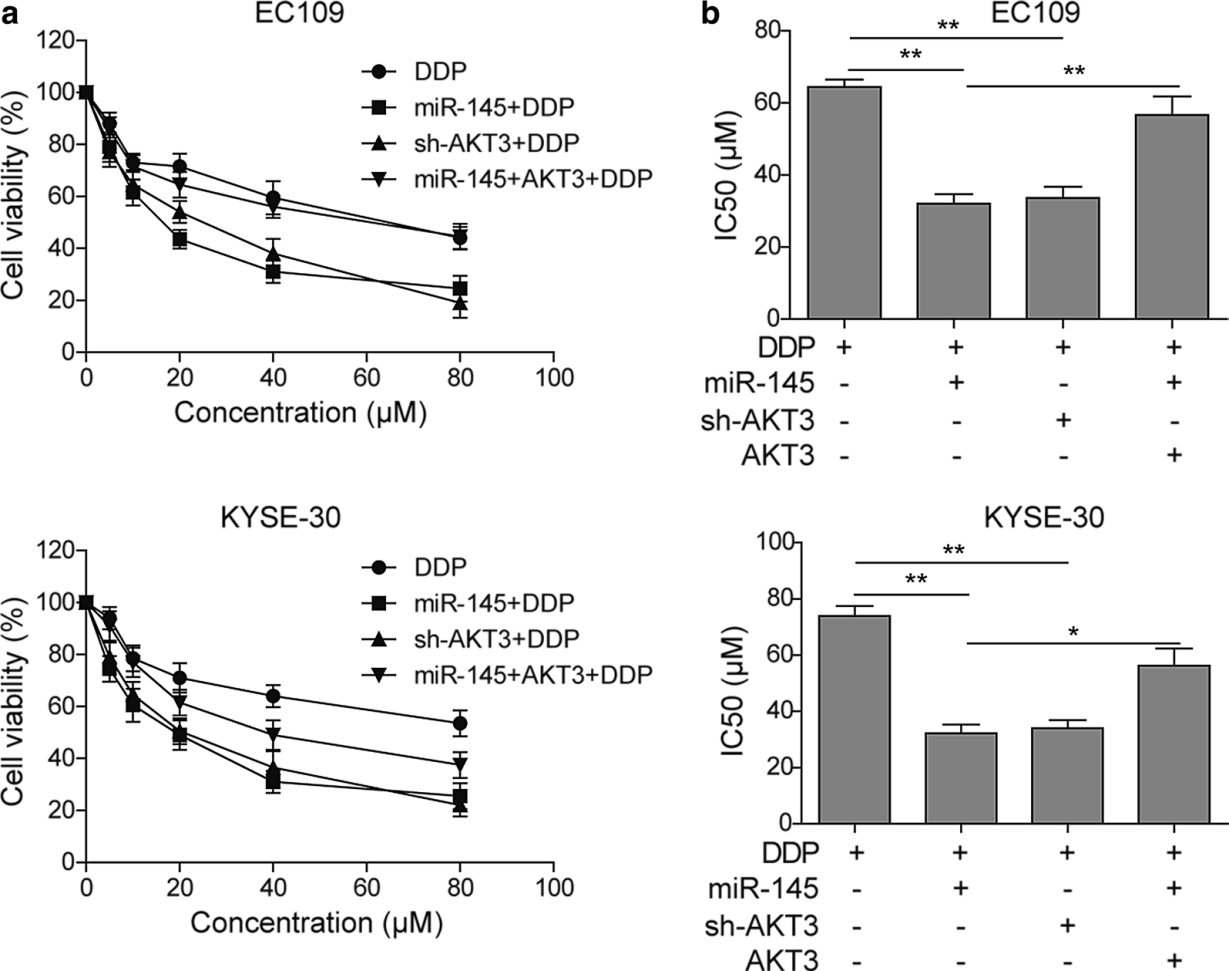
miR-145 sensitized ESCC to DDP through inhibiting AKT3. **a** Cell viability was detected by MTT assay in EC109 and KYSE-30 cells exposed to DDP after transfection with miR-145 mimics, sh-AKT3, or miR-145 mimcis + pcDNA3.1-AKT3. **b** Analysis and quantifications of DDP IC50 value. All the results were shown as mean ± SD (n = 3), which were three separate experiments performed in triplicate. *p < 0.05 and **p < 0.01

To test the effects miR-145 on ESCC chemosensitivity, we assessed cell survival upon DDP treatment. The ESCC cells were treated with DDP at the dose of 5 μM, 10 μM, 20 μM, 40 μM or 80 μM. Compared to the control group, there was a downward shift of the cell viability curve of miR-145 mimics or sh-AKT3 treatment in both EC109 and KYSE-30 cells (Fig. 4a). Then we calculated the IC50 for each group and graphed in Fig. 4b. In EC109, overexpression of miR-145 and knockdown of AKT3 reduced the IC50 of DDP from 64.40 to 32.01 μM and 33.64 μM, respectively. But simultaneous overexpression of miR-145 and AKT3 brought the IC50 back to 56.71 μM. Similarly, overexpression of miR-145 and knockdown of AKT3 reduced the IC50 of DDP by 55.3% and 53.9%, which was abrogated by the co-overexpression of miR-145 and AKT3 in KYSE-30 cells (Fig. 4b). To conclude, miR-145 inhibits the expression of AKT3, rendering ESCC cells more sensitive to DDP.

### miR-145 facilitates DDP-induced cell cycle arrest and apoptosis via targeting AKT3 in ESCC cells

**Fig. 5:**
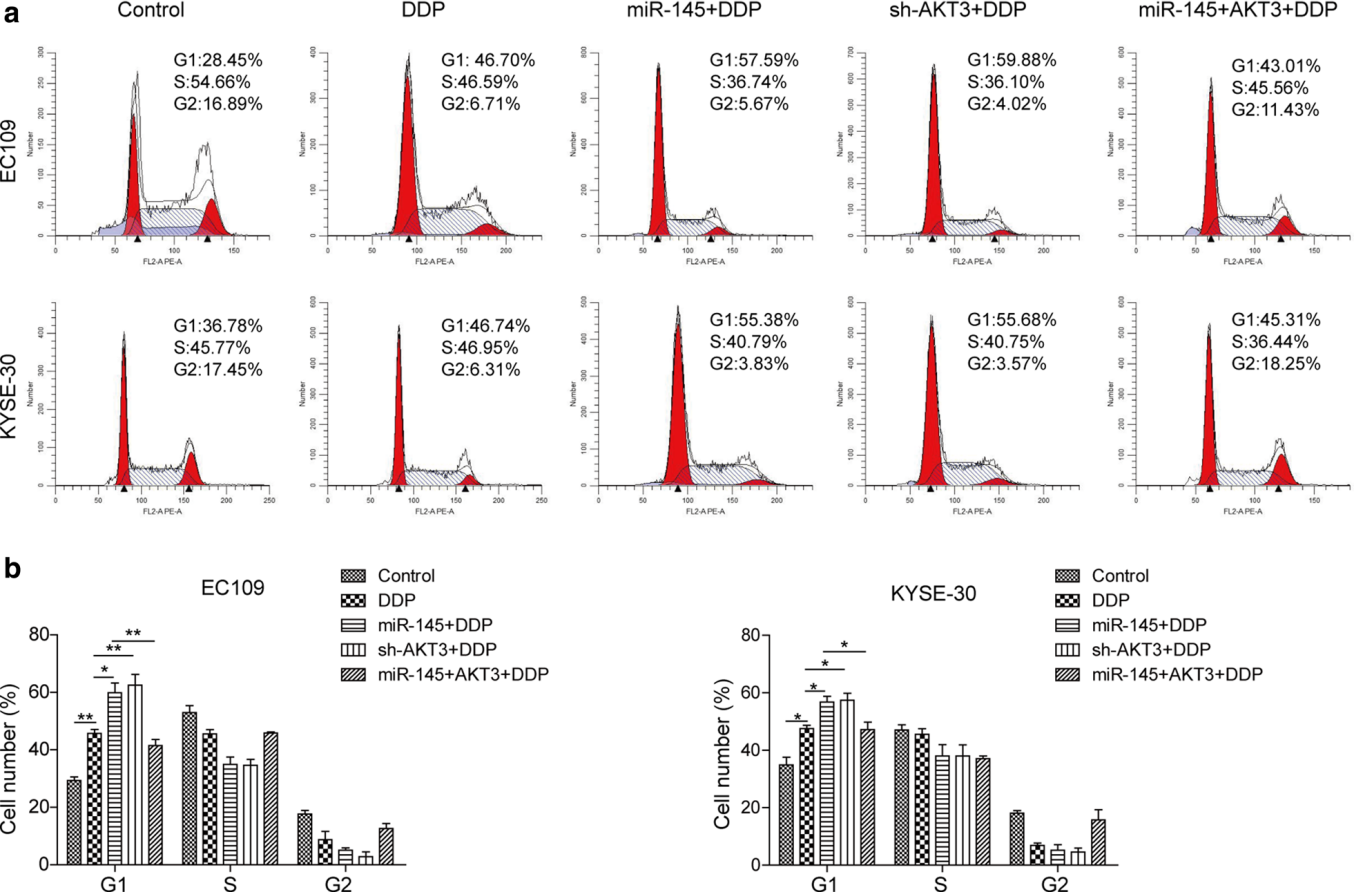
miR-145 facilitated DDP-induced G1 arrest by decreasing AKT3 in ESCC cells. **a** Cell cycle was analyzed with flow cytometry in EC109 and KYSE-30 exposed to DDP after transfection with miR-145 mimics, sh-AKT3, or miR-145 mimcis + pcDNA3.1-AKT3. **b** Statistics of cell number in G1, S and G2 stages. All the results were shown as mean ± SD (n = 3), which were three separate experiments performed in triplicate. *p < 0.05 and **p < 0.01

**Fig. 6:**
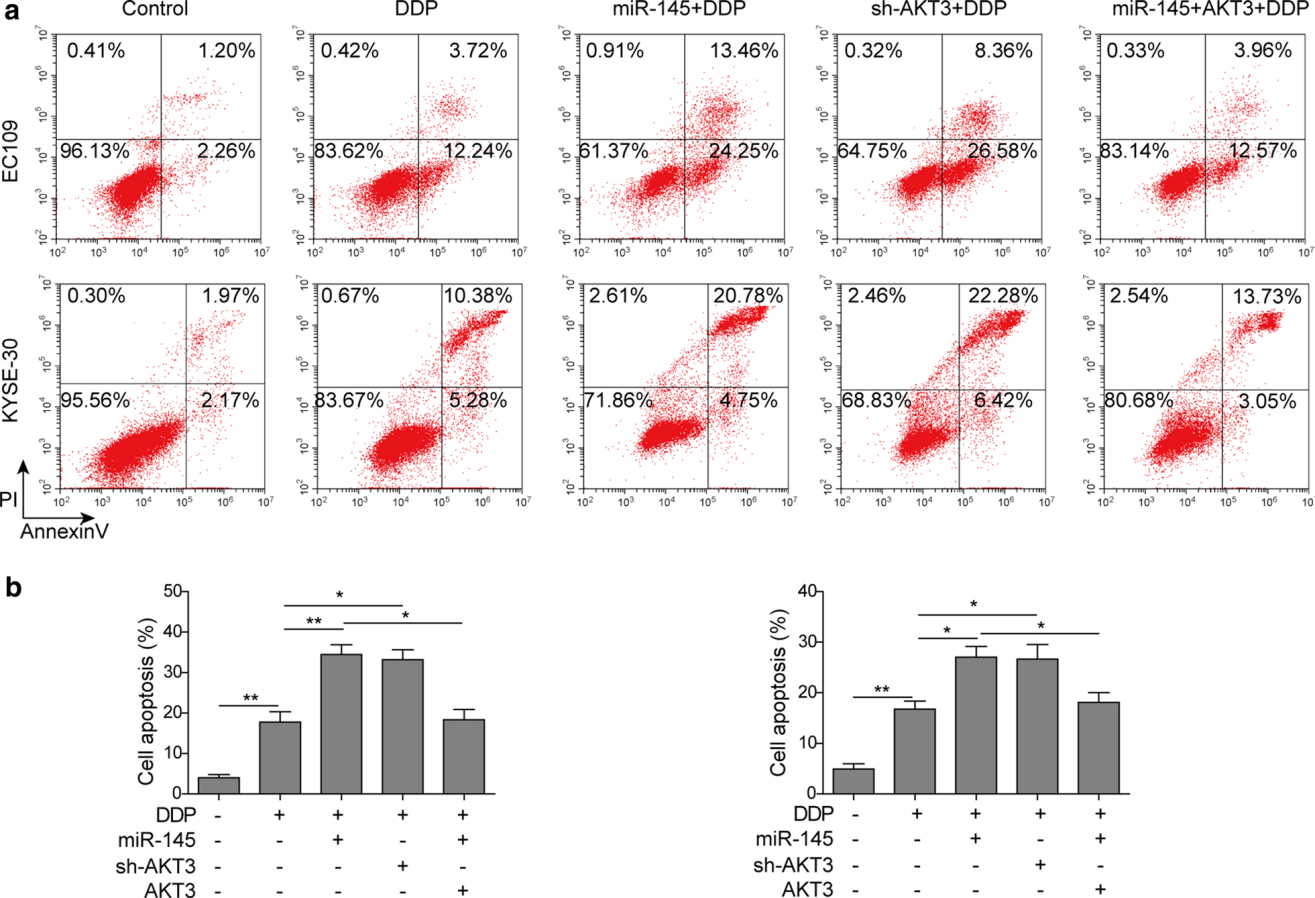
miR-145 increased DDP-induced apoptosis by targeting AKT3 in ESCC cells. **a** Cell apoptosis was analyzed with flow cytometry in EC109 and KYSE-30 exposed to DDP after transfection with miR-145 mimics, sh-AKT3, or miR-145 mimcis + pcDNA3.1-AKT3. **b** Apoptosis cells were quantified. All the results were shown as mean ± SD (n = 3), which were three separate experiments performed in triplicate. *p < 0.05 and **p < 0.01

To verify the biological function of miR-145 and AKT3 on ESCC tumorigenesis, flow cytometry was performed to detect the cell cycle and apoptosis. Cell cycle data demonstrated that ESCC cells transfected with miR-145 mimics or sh-AKT3 had an obvious cell cycle arrest at the G1 phase (Fig. 5a, b). Co-transfection of miR-145 mimics and pcDNA3.1-AKT3 plasmids did not change the cell cycle compared to the control group. Furthermore, as shown in Fig. 6a, b, the miR-145 mimics and sh-AKT3 significantly increased DDP-induced apoptosis in ESCC cells, while co-transfection of miR-145 mimics and pcDNA3.1-AKT3 plasmids had no significant effect on DDP-induced apoptosis. Taken together, miR-145 renders ESCC cells more sensitive to DDP-induced cell cycle arrest and apoptosis.

### miR-145 inhibition desensitizes ESCC cells to DDP treatment in EC9706 cells

**Fig. 7:**
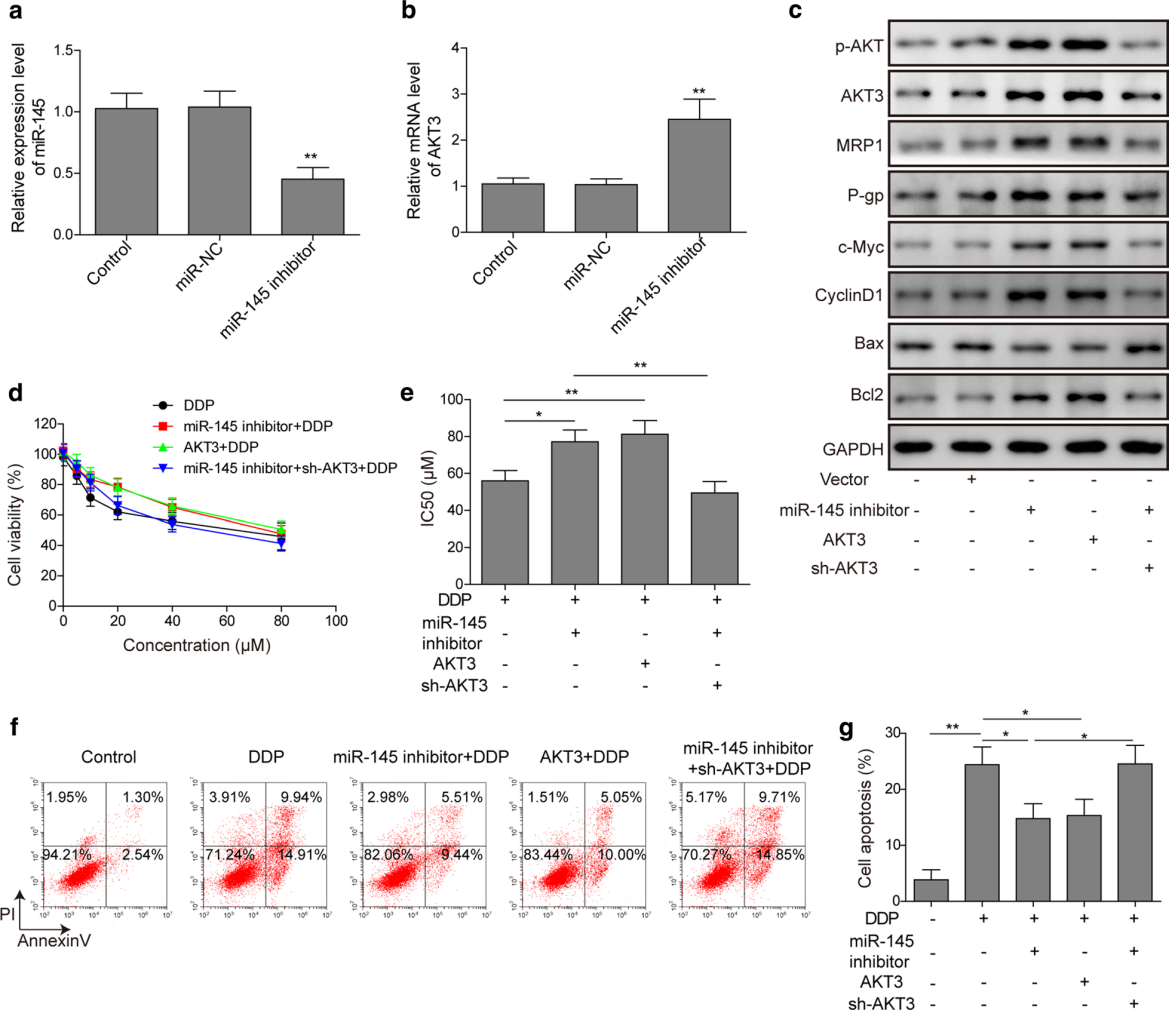
Knockdown of miR-145 inhibited the sensitivity of ESCC cells to DDP by targeting AKT3. The expression level of miR-145 (**a**) and AKT3 (**b**) was detected by qRT-PCR in EC9706 cells transfected with miR-145 inhibitor or miR-NC. **c** The multidrug resistance and cell proliferation-related protein level was detected by Western blotting in EC9706 cells transfected with miR-145 inhibitor, pcDNA3.1-AKT3 or sh-AKT3. **d** Cell viability was detected by MTT assay in EC9706 cells exposed to DDP after transfection with miR-145 inhibitor, pcDNA3.1-AKT3 or sh-AKT3. **e** Analysis and quantifications of DDP IC50 value. **f** Cell apoptosis was analyzed with flow cytometry in EC9706 cells exposed to DDP after transfection with miR-145 inhibitor, pcDNA3.1-AKT3 or sh-AKT3. **g** Apoptosis cells were quantified. All the results were shown as mean ± SD (n = 3), which were three separate experiments performed in triplicate. *p < 0.05 and **p < 0.01

To confirm the role of miR-145 knockdown mediated AKT3 signaling in DDP treatment, EC9706 cell line with high miR-145 level was employed. EC9706 cells were transfected with miR-145 inhibitor, AKT3 overexpression plasmid or AKT3 shRNA plasmids. miR-145 inhibitor significantly repressed miR-145 expression while increased AKT3 mRNA level (Fig. 7a, b). Western blotting revealed that the expression of MRP1, P-gp, c-Myc, cyclin D1 and Bcl-2 was up-regulated while Bax was down-regulated in miR-145 inhibition and AKT3 overexpression group (Fig. 7c and Additional file 2: Fig. S2). Knockdown of miR-145 and overxepression of AKT3 could increase cell viability and the IC50 of DDP in EC9706 cells (Fig. 7d, e). Moreover, miR-145 inhibitor and AKT3 overexpression ameliorated DDP-induced apoptosis dramatically (Fig. 7f, g). However, the efficacy of miR-145 inhibitor was abrogated by co-transfection with AKT3 shRNA (Fig. 7c–g). Taken together, inhibition of miR-145 desensitized ESCC cells to DDP via directly up-regulating AKT3.

### miR-145 increases the inhibition effects of DDP on the growth of xenografts

**Fig. 8:**
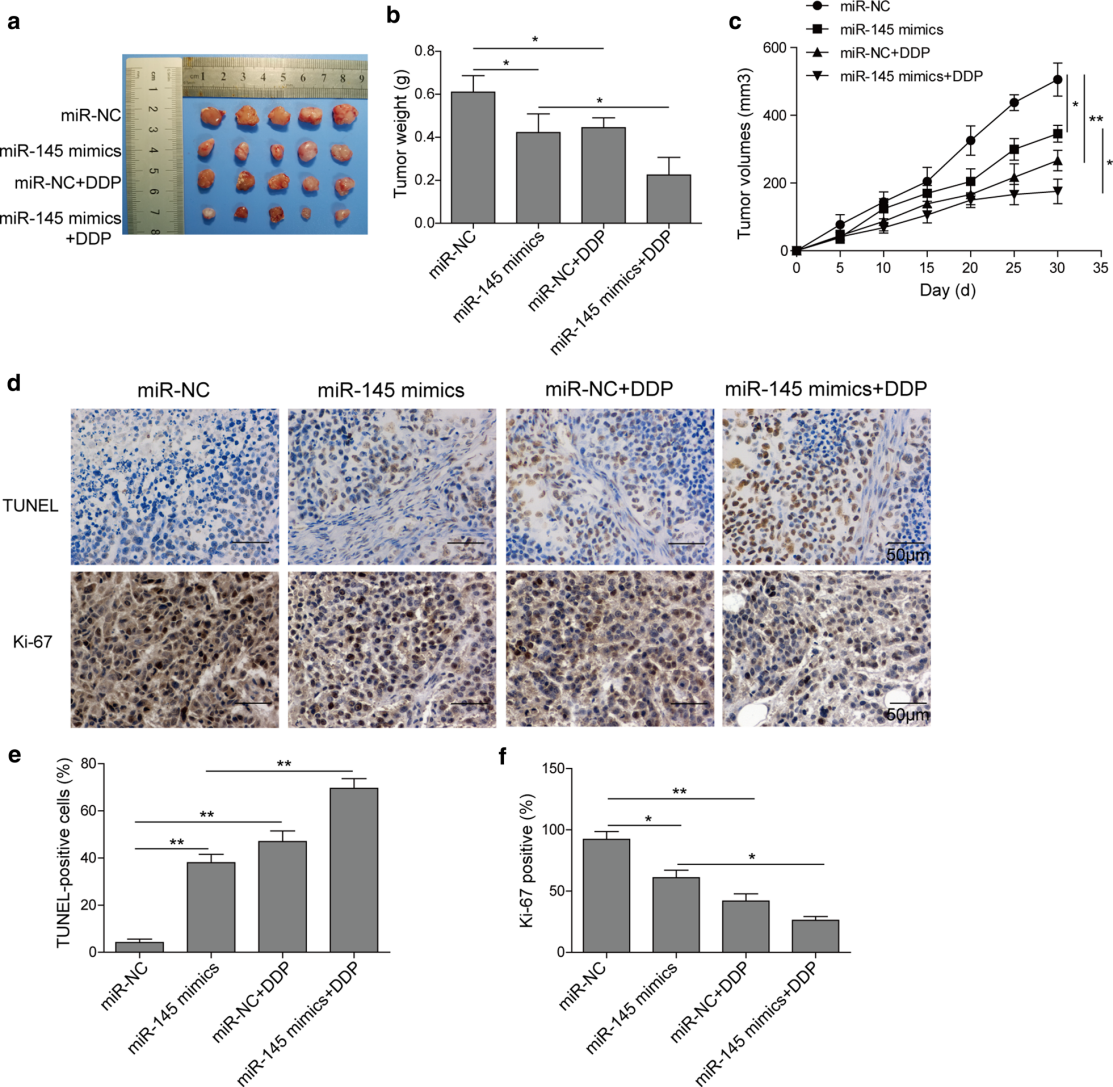
miR-145 improved the inhibition effects of DDP on the growth of xenografts. **a** Tumors from the xenograft treated with different ways for 30 days. **b** The tumor weight from the xenografts. **c** The tumor volume of each group at the end of treatment. **d** Immunohistochemical detection of Ki-67. **e** Quantification of Ki-67 positive rate. All the results were shown as mean ± SD (n = 3), which were three separate experiments performed in triplicate. *p < 0.05 and **p < 0.01

To evaluate the effects of miR-145 in ESCC tumorigenesis in vivo, EC109 were injected subcutaneously into the nude mice. Results revealed that the tumor weight and volume was significantly inhibited by miR-145 mimics, whose efficacy was comparable to DDP treatment (Fig. 8a–c). It is noting that the tumor growth in mice treated with miR-145 mimics was dramatically inhibited by DDP compared to the miR-NC + DDP group. It suggested that miR-145 overexpressed ESCC tissues were more vulnerable to the DDP. Moreover, immunohistochemistry and TUNEL results revealed that in miR-145 overexpression group, the Ki-67 positive rate was lower while TUNEL-positive cells were higher than miR-NC group (Fig. 8d–f). Treatment with miR-145 mimics further decreased Ki-67 level and increased TUNEL-positive cells after DDP administration. Accordingly, these data demonstrated that miR-145 can enhance DDP delivered anti-tumor efficacy in vivo.

## Discussion

ESCC is the most common esophageal carcinoma in China with a high incidence rate [20–22]. Since ESCC patients in early stage demonstrate no obvious symptoms, most ESCC are diagnosed at advanced stage [22]. DDP is widely used in ESCC treatment as the first-line treatment [3, 22, 23]. However, the drug resistance to DDP is frequently occurred, compromising the efficacy of therapy and limiting its clinical use. In past decades, increasing evidences suggested that miRNA play a role in the chemosensitivity of various cancers [23]. An example of these miRNAs is miR-145, whose regulatory role in tumor has been widely explored [24–26]. In the present study, we reported a direct link between miR-145 and AKT3 in ESCC and demonstrated that miR-145 increased the chemosensitivity to DDP through inhibiting multidrug resistance-associated protein MRP1 and P-gp expression by directly suppressing PI3K/AKT signaling pathway. It is necessary to understand the role of miR-145 in the regulation of chemosensitivity and provides a novel molecular target for the efficient clinical therapy.

As a tumor suppressor, aberrant miR-145 expression is discovered in a variety of cancers [14, 15, 27]. Gao et al. [15] reported that miR-145 level decreased in human breast cancer tissues, breast cancer cell lines and doxorubicin resistant MCF-7 cells. Furthermore, miR-145 was down-regulated in colorectal cancer, and overexpression of miR-145 enhanced 5-Fu-induced DNA damage [27]. But Sachdeva et al. [28] demonstrated that up-regulated miR-145 failed to deliver suppression in human breast cancer cell MDA-MB-231 and LM2-4142, indicating that miR-145 exerts its function in a cell-type specific manner. In the present study, we found that down-regulated miR-145 and up-regulated AKT3 are observed in ESCC tissues and cells, implying that miR-145 is tumor suppressor in ESCC. Liu et al. [27] reported miR-145 enhanced 5-Fu efficacy by inhibiting RAD18 and RAD6, DNA damage-activated E3 ubiquitin ligases, through directly targeting RAD18 by interaction with the 3′-UTR in colorectal cancer. Zhu et al. [29] discovered that miR-145 sensitized ovarian cancer cells to the paclitaxel treatment by suppressing the expression of Sp1 and CDK6. This study demonstrated that miR-145 inhibited MRP1 and P-gp expression by binding to AKT3 to inhibit its expression. Overexpression of miR-145 increased the sensitivity of cells to DDP, and restoring AKT3 level in ESCC cells highly expressed miR-145 could increase the IC50 of DDP. Additionally, miR-145 could enhance DDP-mediated anti-tumor efficacy in vivo. These results fully indicated that miR-145 can increase the sensitivity of ESCC to DDP by targeting AKT3. Our study was consistent with previous study that miR-145 could regulate AKT3-mediated PI3K/AKT signaling pathway [14]. However, previous studies were performed in thyroid cancer, mainly to explore the effect of miR-145 on the proliferation and metastasis of thyroid cancer by regulating PI3K/AKT pathway [14]. Here we further confirmed the role of miR-145 in DDP sensitivity of ESCC through AKT3-mediated PI3K/AKT pathway. Furthermore, we found for the first time that AKT3 was a target of miR-145 in ESCC, and overexpression of miR-145 promoted the sensitivity of ESCC to DDP by targeting AKT3 to inhibit PI3K/AKT signaling pathway.

The phosphatidyl-inositol-3-kinase/serine/threonine kinase AKT (PI3K/AKT) signaling pathway is hyper-activated in many human cancers, including glioblastoma, thyroid cancer and ESCC [14, 30, 31]. AKT is a key regulator in this pathway, and is a key therapeutic target in a variety of tumors. AKT participate in the regulation of various tumor progression including cell proliferation, cell metabolism, apoptosis, migration, angiogenesis and chemotherapy resistance [32]. There are three subtypes of AKT called AKT1, AKT2 and AKT3, which have very similar sequences. As reported, inhibition of PI3K/AKT signaling pathway could reversed multidrug resistance through TSC1/2 complex and Rheb in human gastric adenocarcinoma cells [33]. PI3K/AKT signaling pathway was activated in ESCC, and was closely related to the presence of lymph node metastases and advanced TNM stage [31]. However, the relationship between PI3K/AKT signaling pathway and ESCC chemosensitivity is still unclear. In the present study, AKT3 was significantly up-regulating in ESCC tissues and cells, suggesting PI3K/AKT signaling pathway was activated in ESCC. Overexpression of AKT3 and knockdown of AKT3 could promote and inhibit the expression of multidrug resistance and cell proliferation-related proteins including MRP1, P-gp, c-Myc, Cyclin D1 and Bcl-2, respectively. Furthermore, overexpression of AKT3 could inhibit the sensitization of ESCC cells to DDP mediated by miR-145 mimics. These results fully illuminated that miR-145 increases the sensitivity of ESCC cells to DDP by regulating signals downstream of the PI3K/AKT signaling pathway associated with chemosensitivity and proliferation.

miR-145 was reported to regulate cell cycle and apoptosis in numerous tumors. It was reported that miR-145 could induce the cell cycle arrest and inhibit cell proliferation by inhibiting the expression of CDK4 and c-Myc in prostate cancer cells and non-small cell lung cancer cells [34, 35]. Additionally, miR-145 could sensitize ovarian cancer cells to paclitaxel by directly targeting CDK6 to reduce the expression of CDK6, along with downregulation of P-gp [29]. Furthermore, miR-145 inhibited cell proliferation and promoted cell apoptosis through negatively regulating mTOR signaling pathway and decreasing MMP-2 and MMP-9 expression, the Bax/Bcl-2 ratio and the activity of the caspase-3 cascade in human lung adenocarcinoma A549 cells [36, 37]. But how miR-145 regulates cell cycle and apoptosis in ESCC remains elusive. Here, we observed that overexpression of miR-145 and knockdown of AKT3 could promote DDP-induced apoptosis and cycle arrest of ESCC cells. Overexpression of miR-145 could inhibit cell proliferation and anti-apoptosis-related proteins including c-Myc, Cyclin D1 and Bcl-2, while promote pro-apoptosis protein Bax expression by repressing PI3K/AKT signaling pathway.

## Conclusion

In conclusion, our study revealed that miR-145 is an important factor that regulates the sensitivity of ESCC to DDP treatment. miR-145 sensitizes ESCC cells to DDP-induced cell cycle arrest and apoptosis by directly targeting AKT3 to inhibiting PI3K/AKT signaling pathway. Increasing miR-145 enhances the inhibition DDP delivered on tumor growth in vivo. This study provides novel insights on miR-145-dependent regulation of chemoresistance and potential targets in ESCC, suggesting that miRNA-modulating anticancer drugs may be applied in clinical therapy in the future.

## Supplementary information

**Additional file 1: Fig. S1:** Quantitative analysis of protein band gray in Fig. 3d.

**Additional file 2: Fig. S2:** Quantitative analysis of protein band gray in Fig. 7c.

## Data Availability

All data generated or analysed during this study are included in this published article [and its Additional files].
