# Supplementary figures and images for: miR-145 sensitizes esophageal squamous cell carcinoma to cisplatin through directly inhibiting PI3K/AKT signaling pathway
Source: Cancer Cell Int. 2019 Sep 30;19:250. doi: 10.1186/s12935-019-0943-6 (PMC6767650; doi:10.1186/s12935-019-0943-6)

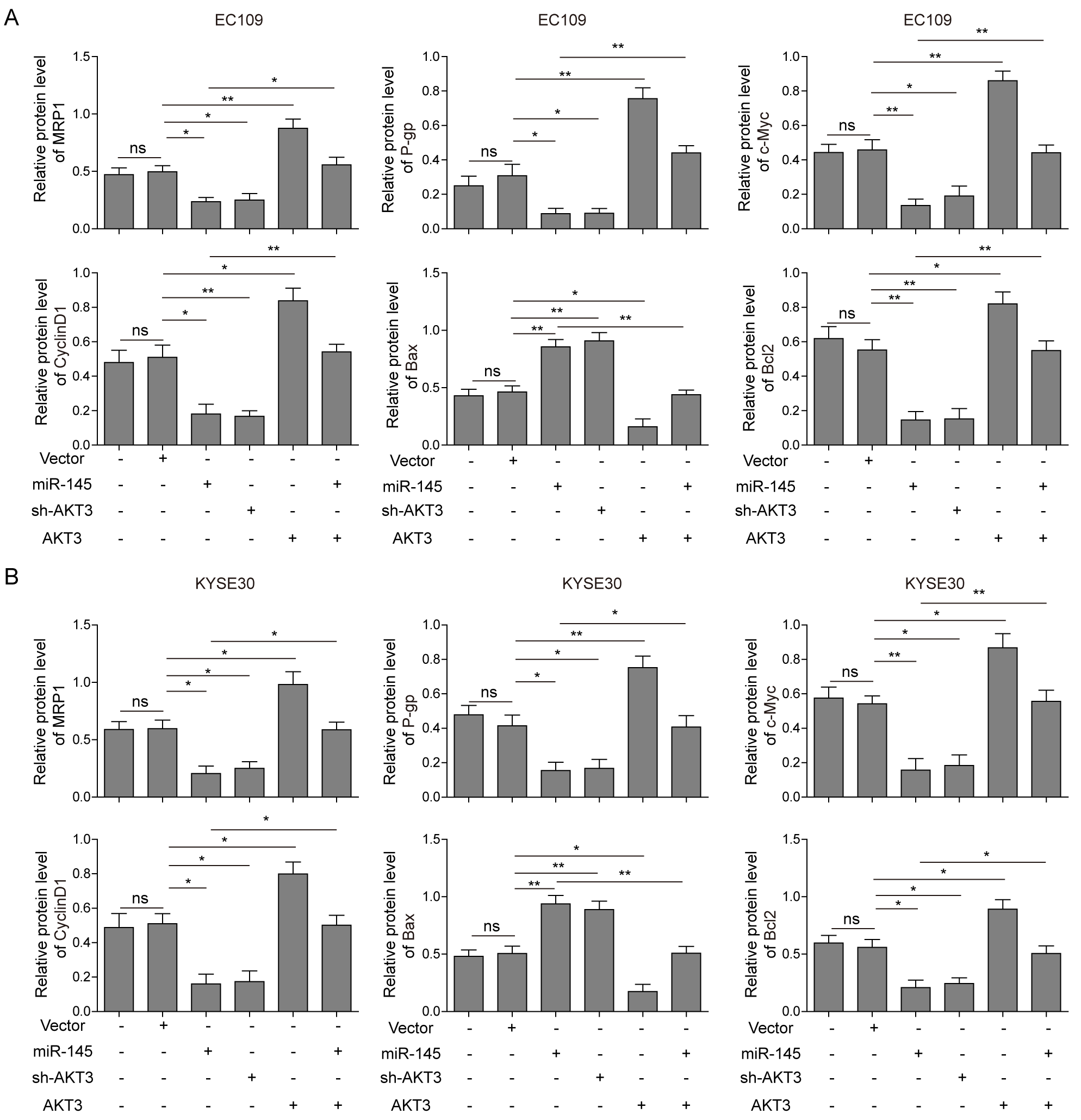

Supplement: Supplementary file 1 — Additional file 1: Fig. S1: Quantitative analysis of protein band gray in Fig. 3d. [file 12935_2019_943_MOESM1_ESM.tif]

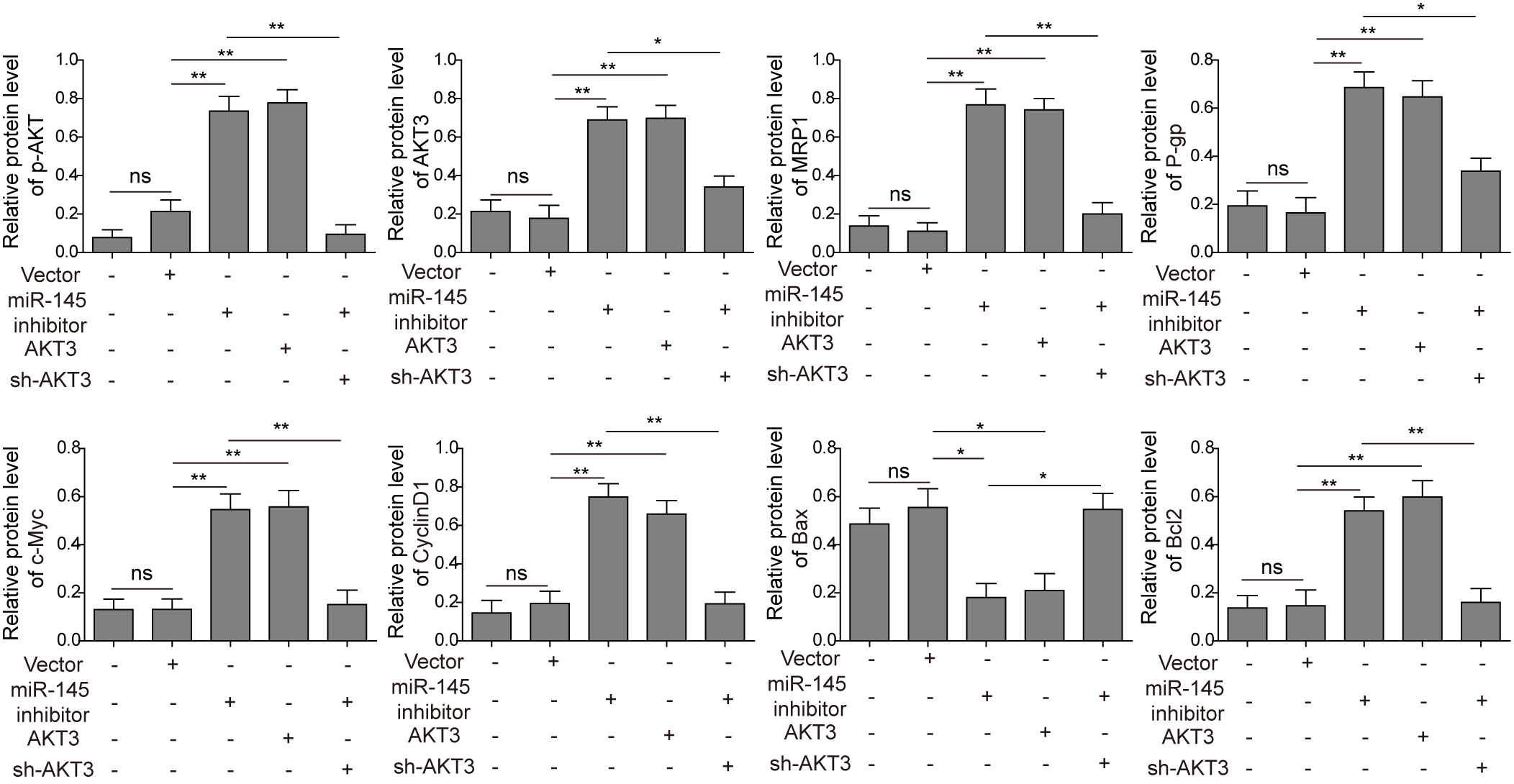

Supplement: Supplementary file 2 — Additional file 2: Fig. S2: Quantitative analysis of protein band gray in Fig. 7c. [file 12935_2019_943_MOESM2_ESM.tif]
